# Characterization of the Rat Oncostatin M Receptor Complex Which Resembles the Human, but Differs from the Murine Cytokine Receptor

**DOI:** 10.1371/journal.pone.0043155

**Published:** 2012-08-22

**Authors:** Johannes Drechsler, Joachim Grötzinger, Heike M. Hermanns

**Affiliations:** 1 From the Rudolf-Virchow-Center, DFG Research Center for Experimental Biomedicine, University of Würzburg, Würzburg, Germany; 2 Institute of Biochemistry, Christian-Albrechts-University of Kiel, Kiel, Germany; Universität Würzburg, Germany

## Abstract

Evaluation of a pathophysiological role of the interleukin-6-type cytokine oncostatin M (OSM) for human diseases has been complicated by the fact that mouse models of diseases targeting either OSM or the OSM receptor (OSMR) complex cannot fully reflect the human situation. This is due to earlier findings that human OSM utilizes two receptor complexes, glycoprotein 130 (gp130)/leukemia inhibitory factor receptor (LIFR) (type I) and gp130/OSMR (type II), both with wide expression profiles. Murine OSM on the other hand only binds to the gp130/OSMR (type II) receptor complex with high affinity. Here, we characterize the receptor usage for rat OSM. Using different experimental approaches (knock-down of the OSMR expression by RNA interference, blocking of the LIFR by LIF-05, an antagonistic LIF variant and stably transfected Ba/F3 cells) we can clearly show that rat OSM surprisingly utilizes both, the type I and type II receptor complex, therefore mimicking the human situation. Furthermore, it displays cross-species activities and stimulates cells of human as well as murine origin. Its signaling capacities closely mimic those of human OSM in cell types of different origin in the way that strong activation of the Jak/STAT, the MAP kinase as well as the PI3K/Akt pathways can be observed. Therefore, rat disease models would allow evaluation of the relevance of OSM for human biology.

## Introduction

The interleukin-6-type cytokine oncostatin M (OSM) was initially described as a cytokine with strong growth inhibitory effects on melanoma cells [1]. Studies over the last decade have, however, suggested that it has pleiotropic activities. Contributions of this cytokine have been identified for hematopoietic progenitor cell homeostasis [2], [3], extrathymic T cell development [4], [5], suppression of fetal liver hematopoiesis [6], [7], liver development [8], [9] and regeneration [10], angiogenesis [11], cardiac remodeling [12] and particularly for inflammatory processes. Elevated expression levels of human OSM are found in inflammatory diseases like rheumatoid arthritis, psoriasis, atherosclerosis [13], [14], [15], [16], [17] and it has been shown to induce inflammatory genes like chemokines [18], [19], [20], [21], [22] or P-selectin [23].

Human OSM (hOSM) is mainly expressed by neutrophils, activated macrophages, dendritic cells and T-cells [1], [17], [24], [25] as a 252 amino acid precursor polypeptide [26]. After cleavage of the N-terminal signal peptide and a C-terminal pro-domain the thus generated mature 196 amino acid protein has been shown to elicit the highest bioactivity [27]. Meanwhile, the bovine, murine and rat OSM orthologs have been cloned [9], [28], [29]. Comparison of the gene organization of OSM with interleukin-6, granulocyte-colony stimulatory factor and leukemia inhibitory factor (LIF) suggested an evolutionary descent from a common ancestral gene [30]. A particularly high homology exists to LIF [31].

So far, the receptor complexes have only been characterized for human and murine OSM (mOSM). Unlike for other IL-6-type cytokines, the receptor systems for OSM differ in composition between man and mouse. Human OSM is able to utilize two receptor complexes: the type I LIFR/gp130 heterodimer and the type II OSMR/gp130 heterodimer [32], [33]. This is in sharp contrast to the murine ortholog which offers high affinity binding sites only for the type II OSMR/gp130 receptor complex [34]. Consequently, in vivo studies carried out in the mouse system cannot correctly address the physiological response to hOSM. Additional information generated by cross-stimulation studies of human and murine cells with OSM originating from both species demonstrated that hOSM can efficiently activate signal transduction in murine cells, however, it utilizes only the type I LIFR/gp130 heterodimer on these cells [34]. Therefore, reconstitution studies using hOSM in mouse models of diseases, which mimic rather LIF than OSM activities, have so far complicated the evaluation of the physiological function of OSM. On the other hand, mOSM is unable to stimulate human cells, a characteristic shared by many other IL-6-type cytokines.

The current study characterizes the receptor complex for rat OSM (rOSM) in order to evaluate the potential of the rat system as more suitable model to evaluate hOSM physiology. Using antagonistic cytokines, RNA interference to block one receptor and stably transfected Ba/F3 cells expressing only one receptor complex at the time, we can show that rOSM indeed uses the type I gp130/LIFR as well as the type II gp130/OSMR complex for signaling. Thereby it closely resembles hOSM. Cross-stimulation studies using human, murine and rat OSM in comparison to LIF further delineate the species-specific receptor usage of the three OSM orthologs.

## Results

### Rat OSM can stimulate human, murine and rat hepatoma cells

Sequence analyses of the mature forms of human, mouse and rat OSM indicate a high degree of sequence and structural homology. Despite this homology, studies carried out by a number of research groups in the last decade have clearly shown that human and murine OSM signal in a species-specific manner: hOSM can signal in human cells via two receptor complexes, the type I gp130/LIFR or the type II gp130/OSMR [32], [33] complex, while mOSM only signals via the type II receptor complex [34]. Additionally, it was shown that hOSM activates only the type I receptor complex (gp130/LIFR) on mouse cells and mOSM fails to activate signaling in human cells [34]. To date the receptor usage of rOSM is unknown.

**Figure 1:**
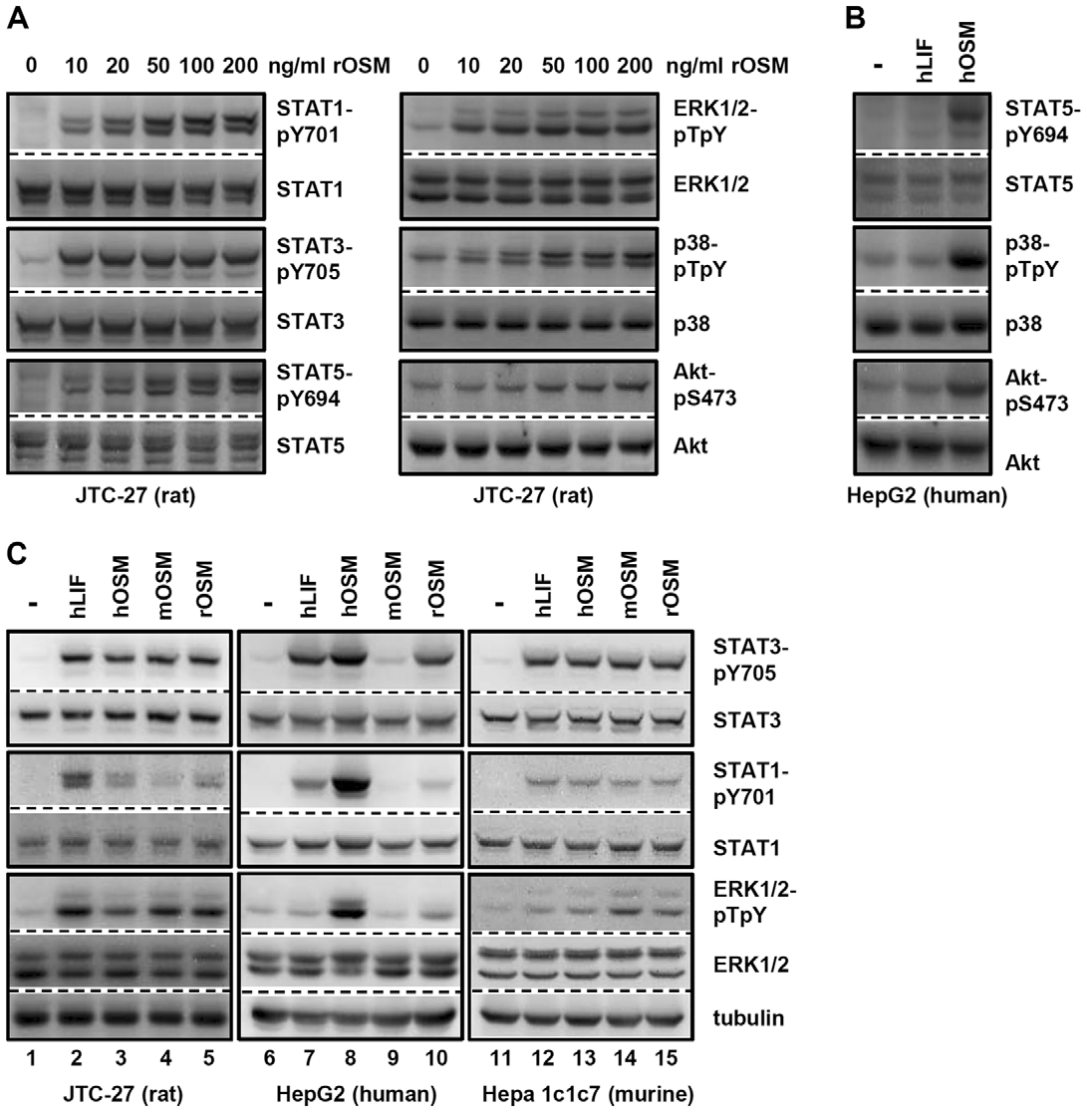
Comparison of human LIF, human, murine and rat OSM induced signal transduction in hepatoma cells. **A**, JTC-27 rat hepatoma cells were treated with the indicated concentrations of rOSM for 15 min. The phosphorylation levels of STAT1, STAT3, STAT5 as well as ERK1/2, p38 and Akt were detected via Western blot analysis. The blots were stripped and reprobed with antibodies recognizing the proteins irrespective of their activation status. **B**, HepG2 human hepatoma cells were exposed to 10 ng/ml hLIF or hOSM for 15 min. Western blots detecting the activation status of the indicated proteins were performed as described in A. **C**, Hepatoma cells from rat (JTC-27), human (HepG2) and murine (Hepa1c1c7) origin were treated with 10 ng/ml hLIF, hOSM, mOSM or rOSM for 15 min. Activation of the indicated proteins was detected via Western blot analysis as described in A. Additionally an α-tubulin loading control was included. Blots shown are representative for 3 or more experiments.

Therefore, we first defined the signaling capacities of rOSM on rat hepatoma cells since they express gp130, LIFR and OSMR (data not shown). Consequently, these cells are capable of forming the type I as well as the type II receptor complexes. Cellular lysates were analyzed for the activation of the Jak/STAT pathway, MAP kinase pathways and PI3K/Akt pathway (Fig. 1A). Regarding the signaling capacities, rOSM turned out to be comparable to hOSM, i.e. it is a strong inducer of the Jak/STAT pathway by activating STAT1, STAT3, STAT5 (Fig. 1A, left panel) and of the ERK1/2 MAPK pathway (Fig. 1A, top right panel). At higher concentrations rOSM additionally activates the MAPK p38 and the survival-promoting PI3K/Akt pathway (Fig. 1A, middle and bottom right panels). In this aspect rOSM equals hOSM which -unlike human LIF (hLIF)- is also a potent inducer of STAT5, p38 and Akt phosphorylation (Fig. 1B).

Murine OSM is known to be unable to stimulate cells of human origin. To address cross-species activities of rOSM we used hepatoma cell lines from rat, mouse and human origin (JTC-27, Hepa1c1c7, HepG2). All three cell lines were stimulated with rat, murine or human OSM (10 ng/ml) as well as hLIF (10 ng/ml) for 15 min. In sharp contrast to mOSM, rOSM can stimulate human hepatoma cells (Fig. 1C, lane 10). It strongly induces the tyrosine phosphorylation of STAT3 and -to a weaker extent- of STAT1. However, it fails to activate ERK1/2 MAPKs. In these aspects, on human cells rOSM mimics the activities of hLIF rather than hOSM (Fig. 1C, compare lanes 7, 8 and 10). On mouse cells, rOSM signals identically to mOSM (Fig. 1C, lanes 14 and 15). Interestingly, mOSM can induce signal transduction on rat hepatoma cells (Fig. 1C, lane 4).

**Figure 2:**
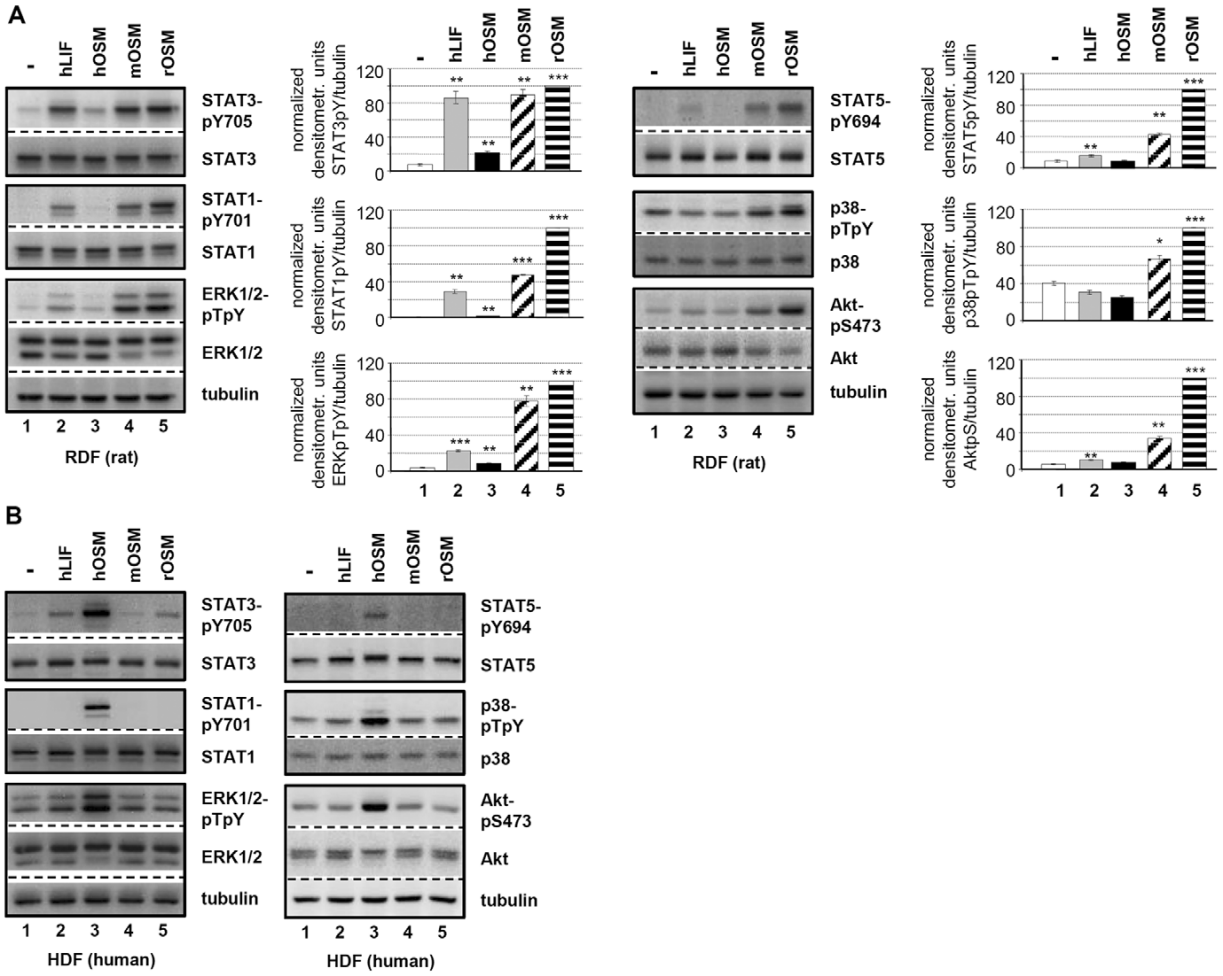
Comparison of hLIF, hOSM, mOSM and rOSM activated signaling pathways in primary dermal fibroblasts. **A**, Rat dermal fibroblasts (RDF) and **B**, human dermal fibroblasts (HDF) were treated with 10 ng/ml hLIF, hOSM, mOSM or rOSM for 15 min. The phosphorylation levels of STAT1, STAT3, ERK1/2 as well as STAT5, p38 and Akt were detected via Western blot analysis. The blots were stripped and reprobed with antibodies recognizing the proteins irrespective of their activation status. Additionally an α-tubulin loading control was included. Phosphorylation intensities were quantified by chemiluminescence analysis and normalized to tubulin. Activation determined for rOSM was set to 100%. Shown are the means (n = 3) with standard error of mean (SEM). * p<0.05, ** p<0.01, *** p<0.001 untreated vs. cytokine-treated sample.

Compared to stimulation of HepG2 with hOSM, the STAT1 activation mediated by rOSM on JTC-27 appeared rather weak, which could indicate a bias of rOSM for STAT3 activation and therefore a potential difference to hOSM. Closer inspection of OSM receptor levels indicated, however, that HepG2 cells express more OSMR than LIFR while in JTC-27 cells higher mRNA levels can be detected for LIFR compared to OSMR (data not shown). The expression level of gp130 is similar in both cell types. Consequently, the ratio of type I to type II receptor complexes differ in the human and rat hepatoma cell line which could be another reason for preferences in STAT activation. Therefore, we additionally stimulated primary dermal fibroblasts from both species with all OSM variants. As shown in Figure 2 no difference is observed between rOSM-mediated signaling in rat dermal fibroblasts (RDF, Fig. 2A) and hOSM-mediated activation of signaling pathways in human dermal fibroblasts (HDF, Fig. 2B). Both OSM variants very potently activate STAT3, STAT1, ERK1/2 (left panels), as well as STAT5, p38 and AKT (right panels) if used at equal concentrations. Identical signaling activities of rOSM are observed in neonatal rat cardiac fibroblasts (Figure S1). Interestingly, on human cells rOSM mimics again hLIF by only activating STAT3. Mouse OSM – as shown before in hepatoma cells – cannot activate signaling in human cells, however, it signals comparably to rOSM on rat cells.

Taken together, rat OSM can stimulate rat, murine and human cells. On rat cells, it is able to activate signaling pathways comparable to human OSM on human cells.

### Rat OSM signals through the type I and type II receptor complex on rat hepatoma cells

In order to characterize the receptor complexes used by rOSM on rat hepatoma cells, we performed RNA interference studies to abrogate the expression of the rat OSMR or blocked the rat LIFR by a LIFR-specific antagonist (LIF-05, [35]).

**Figure 3:**
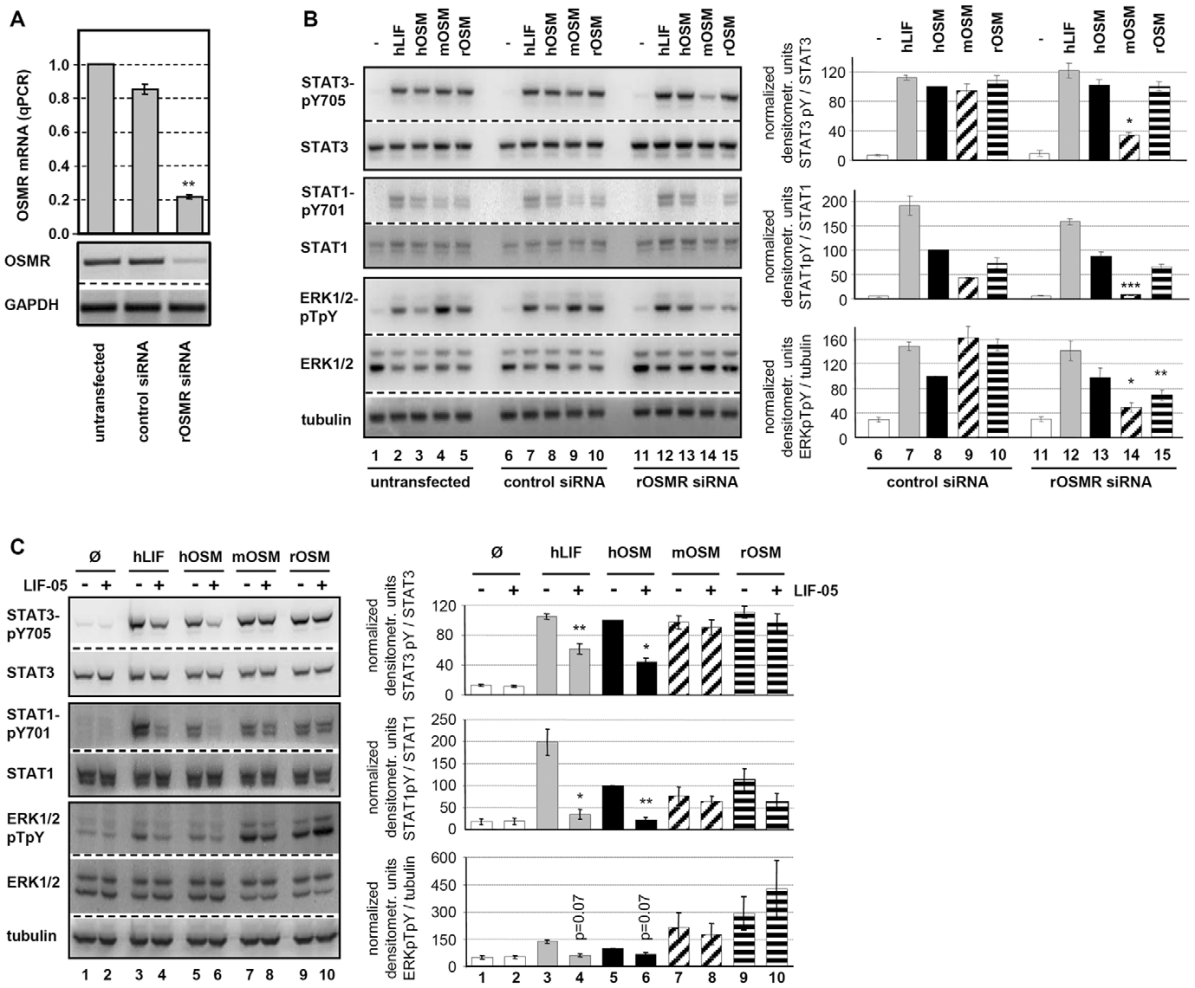
OSMR RNA interference and LIFR antagonistic blockade elucidate rOSM receptor preference. **A**, JTC-27 were transfected with control or rOSMR siRNA or left untransfected. Transfection efficiencies were analyzed by quantitative real time RT-PCR (top) and semiquantitative RT-PCR (bottom). **B**, Untransfected, control siRNA and rOSMR siRNA transfected JTC-27 were treated with 10 ng/ml hLIF, hOSM, mOSM and rOSM for 15 min. Lysates were subjected to Western blot analysis using antibodies specific for the indicated proteins. The blots were stripped and reprobed with antibodies recognizing the proteins irrespective of their phosphorylation status and with an α-tubulin antibody. Phosphorylation intensities were quantified by chemiluminescence analysis and normalization to loading controls. Activation determined for hOSM was set to 100%. Shown are the means (n = 3) with standard error of mean (SEM). * p<0.05, ** p<0.01, *** p<0.001 OSMR siRNA vs. control siRNA. **C**, JTC-27 were preincubated with LIF-05 (50 ng/ml, 30 min) and subsequently stimulated with 10 ng/ml hLIF, hOSM, mOSM and rOSM for 15 min. The phosphorylation intensities of indicated proteins were detected via Western blot analysis. Loading controls and quantification of chemiluminescence intensities were performed as described in B. Shown are the means (n = 3) with standard error of mean (SEM). * p<0.05, ** p<0.01 untreated vs. LIF05-pretreated sample.

Transfection of JTC-27 rat hepatoma cells with siRNA targeting the rat OSMR resulted in a reduction of OSMR mRNA levels by 80% (Fig. 3A). Specificity of the knock-down was confirmed by stimulation of siRNA-transfected cells with hLIF. This stimulation resulted in comparable phosphorylation of STAT1, STAT3 and ERK1/2 in OSMR siRNA-transfected, control siRNA-transfected or untransfected cells (Fig. 3B, lanes 2, 7, 12; quantification for control and OSMR siRNA). Thereby we could exclude that gp130 or the LIFR were affected by the OSMR siRNA since LIF signals exclusively via the gp130/LIFR complex. Furthermore, no changes in protein levels for any of the signaling molecules analyzed could be detected.

Interestingly, the activation of STAT3 and STAT1 in response to rOSM was not significantly affected by rOSMR knock-down (Fig. 3B, 1^st^ and 2^nd^ panel, lane 15). However, a strong reduction in signaling was observed for ERK1/2 for which the phosphorylation level dropped by more than 50% (Fig. 3B, 3^rd^ panel, lane 15). This is in sharp contrast to murine OSM. Signal transduction in response to mOSM was reduced by up to 80% in all pathways analyzed, i.e. STAT3, STAT1 and ERK1/2 phosphorylation (Fig. 3B, lane 14). This correlates very well with the knock-down efficiency of the OSMR (reduction of 80%, Fig. 3A). Human OSM on the other hand was not affected at all by the knock-down of the rat OSMR (Fig. 3B, lane 13).

Therefore, these results gave first hints that rat OSM -in contrast to murine OSM- can use the LIFR to transmit signals into cells and most likely uses two signaling receptor complexes on rat cells (rgp130/rOSMR, rgp130/rLIFR). Murine OSM uses the type II gp130/OSMR and human OSM utilizes the type I gp130/LIFR complex on rat cells.

To verify this hypothesis, the usage of the rat LIFR was blocked by the LIFR antagonist LIF-05. This protein represents a mutein of LIF in which the binding site for the LIFR (site 3) is maintained while the binding site for gp130 (site 2) is destroyed by site-directed mutagenesis. It has been shown that this LIF variant binds to the LIFR, but since it cannot bind to gp130 serves as a potent antagonist [35]. We verified this antagonistic activity of LIF-05 by showing that it strongly impairs the signaling capabilities of LIF on JTC-27 cells (Fig. 3C, compare lanes 3 and 4). Similarly, signaling by human OSM is strongly impaired by pretreatment of JTC-27 cells with LIF-05 (Fig. 3C, lanes 5 and 6). This confirms the before mentioned observation that human OSM utilizes exclusively the type I gp130/LIFR complex on rat cells which is equivalent to its behavior on murine cells.

As hypothesized, activation of STAT3, STAT1 or ERK1/2 by rOSM was hardly negatively affected by blockade of the rLIFR (Fig. 3C, lanes 9 and 10). This clearly verifies that -in absence of binding sites to rLIFR- rOSM can signal via activation of the gp130/OSMR complex. The increase in ERK1/2 activation upon rOSM stimulation of LIF-05-treated hepatoma cells (Fig. 3C, lane 10) indicated that the OSMR offers higher affinity binding sites for the activation of this MAPK pathway compared to the LIFR. Since murine OSM has no known affinity for LIFR, LIF-05 was without any effect on the signal transduction by mOSM (Fig. 3C, lane 7 and 8).

**Figure 4:**
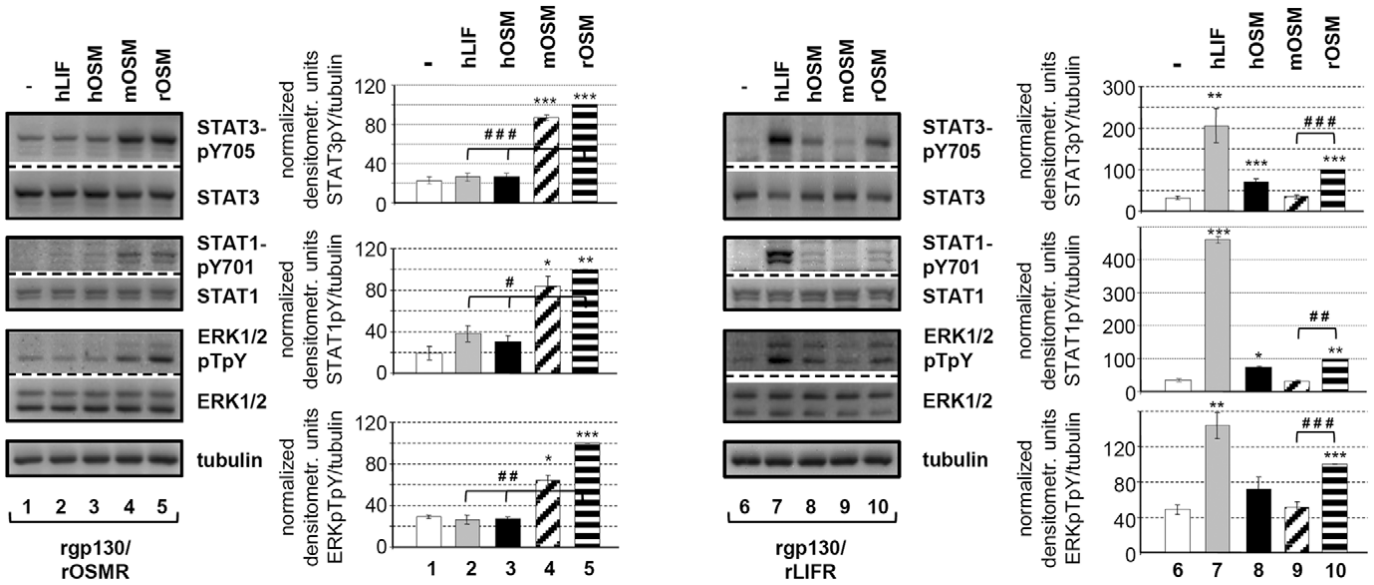
Verification of dual receptor usage by rOSM in stably transfected Ba/F3 cells. Ba/F3 cells stably expressing rgp130/rLIFR or rgp130/rOSMR were treated with 10 ng/ml hLIF or 20 ng/ml hOSM, mOSM or rOSM for 15 min. Lysates were subjected to Western blot analysis using antibodies specific for the indicated proteins. Loading controls were performed as described in 3B. Phosphorylation intensities were quantified by chemiluminescence analysis and normalized to tubulin. Activation determined for rOSM was set to 100%. Shown are the means (n = 3 for STAT1 and ERK, n = 6 for STAT3) with standard error of mean (SEM). * p<0.05, ** p<0.01, *** p<0.001 untreated vs. cytokine-treated sample, # p<0.05, ## p<0.01, ### p<0.001 for rOSM vs. either hLIF/hOSM or mOSM.

In order to provide irrevocable evidence for the above mentioned findings that rOSM uses two receptor complexes on rat cells, we cloned rgp130, rLIFR and rOSMR from transcripts extracted from the rat hepatoma cells (JTC-27). The combinations rgp130/rLIFR (type I) and rgp130/rOSMR (type II) were stably expressed in murine Ba/F3 cells. This pre B-cell line is known to be devoid of expression of gp130, LIFR or OSMR and is therefore the perfect model cell line to analyze the signaling capacity of either rgp130/rLIFR or rgp130/rOSMR in response to rOSM stimulation. Indeed, rgp130/rOSMR as well as rgp130/rLIFR expressing pools of Ba/F3 cells turned out to be responsive to rOSM (Fig. 4). Interestingly, both receptor complexes allowed statistically significant activation of the transcription factors STAT3 and STAT1 (Fig. 4, upper and middle panel, lanes 5 and 10) as well as of the MAP kinases ERK1/2 (Fig. 4, lower panel, lanes 5 and 10). As expected, murine OSM was unable to stimulate the rgp130/rLIFR complex (Fig. 4, lane 9), while it strongly induces signaling downstream of the rgp130/rOSMR complex (Fig. 4, lane 4). On the other hand, human OSM activates only the rat gp130/LIFR complex, but not rgp130/rOSMR (lanes 3 and 8).

Taken together, our data indubitably demonstrate that rat OSM has the capability to activate the type I rgp130/rLIFR as well as the type II rgp130/rOSMR receptor complex. Thereby, its binding properties are equivalent to those of the human OSM on human cells and differ substantially from the murine ortholog.

### Rat OSM utilizes mainly the type II receptor complexes on murine cells

As shown in Figure 1 rOSM can induce signal transduction in murine cells, and is therefore comparable to hOSM. From hOSM it is known that it only utilizes the type I mgp130/mLIFR receptor complex on mouse cells [34]. In order to determine whether the same is true for rOSM, we transfected the murine hepatoma cell line Hepa1c1c7 with siRNA targeting murine OSMR mRNAs.

**Figure 5:**
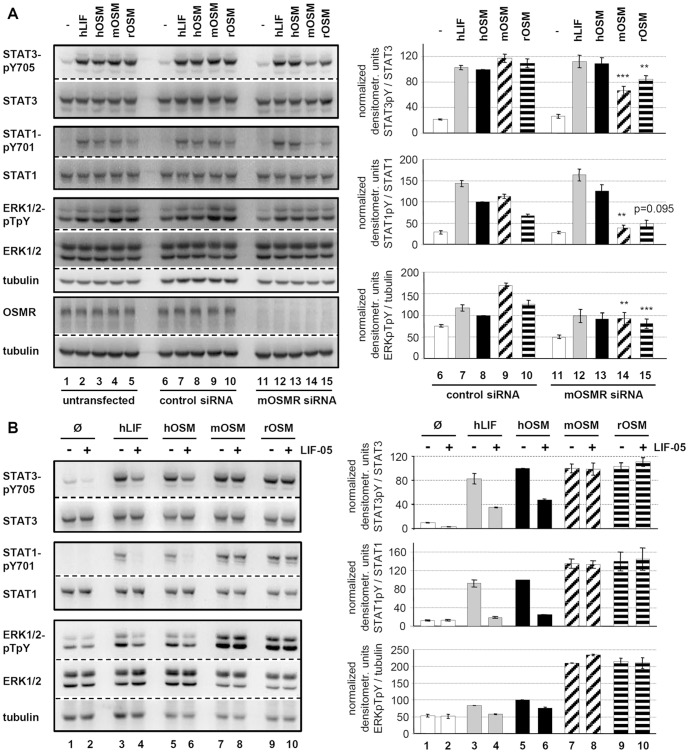
Effects of OSMR knock-down and LIFR blockade for the receptor preference of LIF and OSM in murine cells. **A**, Untransfected, control and mOSMR siRNA-transfected Hepa1c1c7 cells were treated with 10 ng/ml hLIF, hOSM, mOSM and rOSM for 15 min. Phosphorylation levels of the indicated proteins as well as quantification were detected as described in legend to Figure 3B.* p<0.05, ** p<0.01 and *** p<0.001 OSMR siRNA vs. control siRNA (n = 4). **B**, Hepa1c1c7 were, as indicated, pre-incubated with LIF-05 (50 ng/ml, 30 min) and subsequently stimulated with 1 ng/ml hLIF, 10 ng/ml hOSM, mOSM or rOSM for 15 min. The phosphorylation intensities of the indicated proteins were analyzed and quantified as described in legend to Figure 3B (n = 2).

Knock-down efficiencies similar to the rat OSMR could be achieved (Fig. 5A, 4^th^ panel). When we analyzed the signaling capacities of rat, murine and human OSM, we realized that Hepa1c1c7 cells displayed a high basal ERK1/2 phosphorylation which was not abrogated by serum starvation. Therefore, hOSM as well as hLIF only weakly increased the basal ERK1/2 phosphorylation, which -as expected- was not reduced by mOSMR knock-down. Indeed, none of the hLIF or hOSM-induced signaling pathways was significantly reduced by knock-down of the murine OSMR (Fig. 5A, compare lanes 7, 8 with lanes 12, 13).

Murine and rat OSM, however, clearly increased ERK phosphorylation and knock-down of mOSMR expression almost completely abrogated the induced increase in ERK phosphorylation (Fig. 5A, 3^rd^ panel, compare lanes 9, 10 with lanes 14, 15). This indicates that the rodent OSM variants induce ERK activation via usage of the type II gp130/OSMR complex. Regarding the STAT activation, we can clearly show that STAT1 tyrosine phosphorylation is also mediated by the type II receptor complex in response to rodent OSMs since it is severely impaired upon OSMR knock-down (Fig. 5A, 2^nd^ panel). STAT3 activation is also significantly reduced, however, it appears that the low residual expression of the OSMR is sufficient to still allow decent STAT3 activation (Fig. 5A, 1^st^ panel).

Blockade of the murine LIFR by LIF-05 confirmed these findings since only the signal transduction initiated by hLIF and hOSM is strongly reduced (Fig. 5B, lanes 3 vs. 4 and 5 vs. 6), while both rodent versions of OSM fully transduce their signals (Fig. 5B, lanes 7 vs. 8 and 9 vs. 10).

### Rat OSM utilizes mainly the type I receptor complexes on human cells

**Figure 6:**
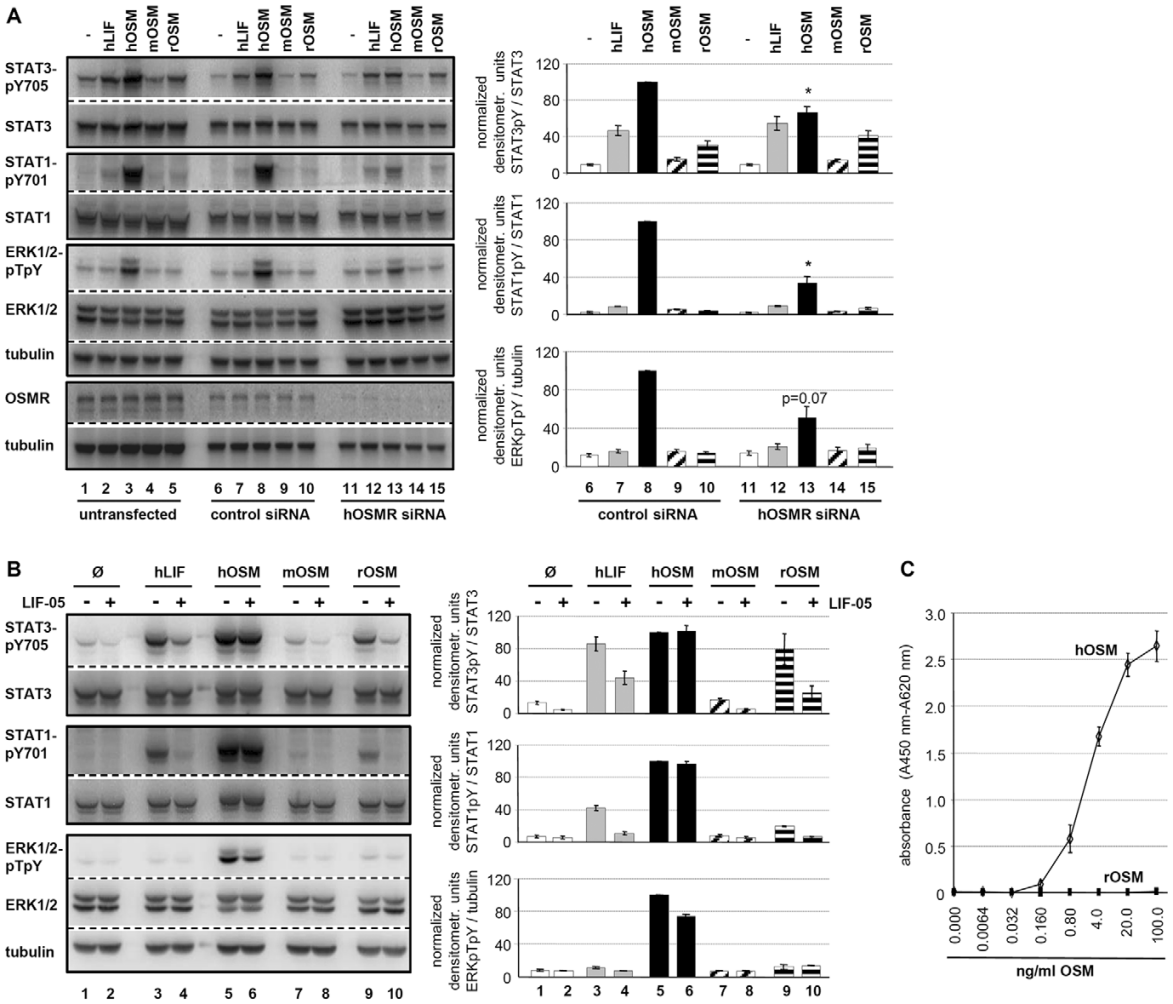
Alterations of LIF- and OSM-induced signaling upon hOSMR knock-down or inhibition of LIFR-gp130 dimerization in HepG2. **A**, Untransfected, control and hOSMR siRNA transfected HepG2 cells were exposed to 10 ng/ml hLIF, hOSM, mOSM or rOSM, respectively, for 15 min. Whole cell lysates were subjected to Western blot analysis and levels of activated STAT3, STAT1 and ERK1/2 were detected and quantified as described in legend to Figure 3B. Shown are the means with standard error of mean (SEM). * p<0.05 OSMR siRNA vs. control siRNA (n = 3 for STAT3, ERK1/2 and hOSM-treated STAT1, n = 2 for hLIF, mOSM, rOSM-treated STAT1). **B**, HepG2 were pre-incubated with LIF-05 (50 ng/ml) for 30 min and treated with 10 ng/ml hLIF, hOSM, mOSM and rOSM for additional 15 min. The phosphorylation intensities of the indicated proteins were analyzed and quantified as described in legend to Figure 3B (n = 2). **C**, Ba/F3 cells stably expressing hgp130 and hOSMR were treated with hOSM or rOSM (0.0064–100 ng/ml) for 48 h. Afterwards, WST-1 reagent was added for 4 h. Values shown represent means (± SEM) of absorbance measurements at 450 nm minus absorbance at 660 nm (n = 3).

As mentioned before, rat OSM differs substantially from murine OSM since it can 1) utilize two receptor complexes and 2) stimulate cells of human origin. Performing equivalent experiments as before by either knock-down of the human OSMR or blockade of the human LIFR by LIF-05 treatment, we clarified the receptor usage for rat OSM on human cells. Knock-down of the human OSMR by siRNA (Fig. 6A, 4^th^ panel) did not negatively affect rOSM-mediated signaling; it rather led to a slight increase in rOSM-mediated STAT3 activation (Fig. 6A, 1^st^ panel, lane 15). Vice versa, blockade of the hLIFR by LIF-05 completely abrogated rOSM-mediated STAT1 and STAT3 activation (Fig. 6B, compare lanes 9 and 10). Therefore, unlike in rat or murine cells, rat OSM exclusively utilizes the hgp130/hLIFR type I receptor complex in human cells.

To show that rat OSM completely lacks affinity for the human OSMR, we stimulated Ba/F3 cells expressing exclusively the type II receptor complex of hgp130/hOSMR with rat and human OSM. While human OSM can induce proliferation of these cells in doses as low as 0.8 ng/ml with saturation at 20 ng/ml, rat OSM was unable to induce proliferation of Ba/F3-hgp130/hOSMR cells irrespective of the concentration used (Fig. 6C).

## Discussion

The interleukin-6-type cytokine oncostatin M is well known to be secreted by activated neutrophils, macrophages, dendritic cells as well as T cells [1], [17], [24], [25] and elevated expression levels of this cytokine have been determined in many inflammatory diseases [13], [14], [15], [16]. Its receptor complexes, gp130/LIFR and gp130/OSMR, are known to be expressed on a wide variety of cells of different origin. Its physiological function, however, is still unclear and controversially discussed.

For example, during inflammation OSM has been attributed pro- as well as anti-inflammatory activities. Administration of recombinant human OSM to LPS-pretreated mice strongly reduced the LPS-induced TNFα secretion and prolonged the survival of these animals [36]. Furthermore, the degree of joint destruction was reduced in these mice indicative of an anti-inflammatory activity of OSM [36]. On the other hand, intra-articular administration of adenoviral-encoded OSM strongly induced a rheumatoid arthritis-like phenotype in mice [37] and administration of neutralizing antibodies against OSM strongly attenuated the symptoms of collagen- and pristane-induced arthritis arguing for a strong pro-inflammatory role [38]. Similarly, inhalation of adenovirus particles encoding mOSM resulted in exacerbated infiltration of eosinophils into the lung of infected mice [19].

One explanation for these controversial findings might originate from the fact that OSM derived from different species was used to stimulate mouse cells. The study claiming an anti-inflammatory role of OSM made use of recombinant human OSM injected into mice [36] while a number of studies pointing to a more pro-inflammatory role administered murine OSM in murine cells [19], [37], [38]. On the molecular level this usage of OSM from different species results in the stimulation of different receptor complexes: human OSM exclusively binds to the type I gp130/LIFR system in mouse cells; murine OSM, however, exclusively activates the type II gp130/OSMR system. Indeed, a recent study with mice overexpressing bovine, human and murine OSM by retroviral gene transfer confirmed this receptor usage and demonstrated that mice overexpressing bovine or human OSM displayed a LIF-like phenotype, while murine OSM overexpressing mice differed significantly in their phenotype [39]. Strictly speaking, none of these studies was able to analyze a situation like it is found in the human system, in which OSM uses both receptor systems. Actually, all three mouse models exhibit rather mild phenotypes which are in sharp contrast to all studies applying retroviral or adenoviral OSM or all in vitro studies which showed exacerbated inflammatory gene expression upon OSM stimulation. Therefore, there is a demand for animal models reflecting the human situation more precisely.

This study provides evidence that rat OSM is identical to human OSM with respect to its receptor usage: like the human ortholog rat OSM has the capability to signal via both, the type I gp130/LIFR as well as the type II gp130/OSMR receptor complex. Knockdown of the rat OSMR by more than 80% has almost no effect on the STAT1 or STAT3 activation by rOSM in rat hepatoma cells (Fig. 3B) which is indicative of the gp130/LIFR usage in absence of available OSMR. This is in sharp contrast to the mouse ortholog, for which knockdown of the OSMR almost completely abrogates signaling (Fig. 3B). Interestingly, activation of the MAP kinases ERK1 and ERK2 in response to rOSM is much more severely affected by the knock-down of the OSMR than the activation of the STAT transcription factors. This led us to hypothesize that the LIFR offers only much lower affinity binding sites for adapter molecules linking the receptor to MAPK activation.

On the other hand, rOSM also appears to use the gp130/OSMR complex since blockade of the LIFR binding sites by the mutant LIF protein LIF-05, which still binds the LIFR with high affinity via its site 3, but cannot bind gp130 due to point mutations in the site 2 of the cytokine [35], does not affect the signaling capacity of rat OSM (Fig. 3C). The efficiency of LIF-05 in blocking access to the LIFR for other cytokines was proven by the finding that STAT as well as ERK activation in response to both, LIF itself as well as hOSM is strongly impaired upon pretreatment of rat hepatoma cells with LIF-05 (Fig. 3C). Interestingly, blockade of the LIFR by LIF-05 resulted in an even slightly enhanced ERK activation in response to rOSM (Fig. 3C, quantification right panel). Therefore, forcing the cytokine into a type II receptor usage appears to strengthen activation of the ERK MAPK cascade. The OSMR appears to be a more potent activator of this pathway which might be due to the conserved Shc adapter binding site (Y861 in hOSMR) in the cytoplasmic region. This tyrosine motif and the Shc adapter protein were shown to be important for the OSMR-mediated activation of the MAPK pathway in response to human OSM [40]. The LIFR requires the tyrosine phosphatase SHP-2 for the activation of ERK1/2 which besides acting as adapter molecule might also perform strong negative regulatory function due to its phosphatase activity [41], [42].

Generation of stably transfected Ba/F3 cells which only express the rat type I rgp130/rLIFR or the rat type II rgp130/rOSMR complex gave doubtless proof that rat OSM displays high affinity for both receptor complexes (Fig. 4).

Further characterization of the receptor usage of rOSM on cells of other species origin led to the finding that rOSM can only use the gp130/LIFR type I receptor complex on human cells. Effective signal transduction in human hepatoma cells was clearly observed (Fig. 1C, 6A, 6B), however, blockade of the hLIFR abrogated signaling (Fig. 6B) and stably transfected Ba/F3 cells expressing only the hgp130/hOSMR combination were unable to grow in response to rOSM (Fig. 6C).

On mouse cells rOSM appears to mainly utilize the type II mgp130/mOSMR complex. Knockdown of the murine OSMR did result in a strong reduction of the OSMR mRNA and protein levels (more than 80%), consequently, signaling of mOSM, but also of rOSM, was significantly reduced (Fig. 5A). On the other hand, blockade of the mLIFR by LIF-05 did not affect rat OSM signaling at all on mouse cells (Fig. 5B).

**Figure 7:**
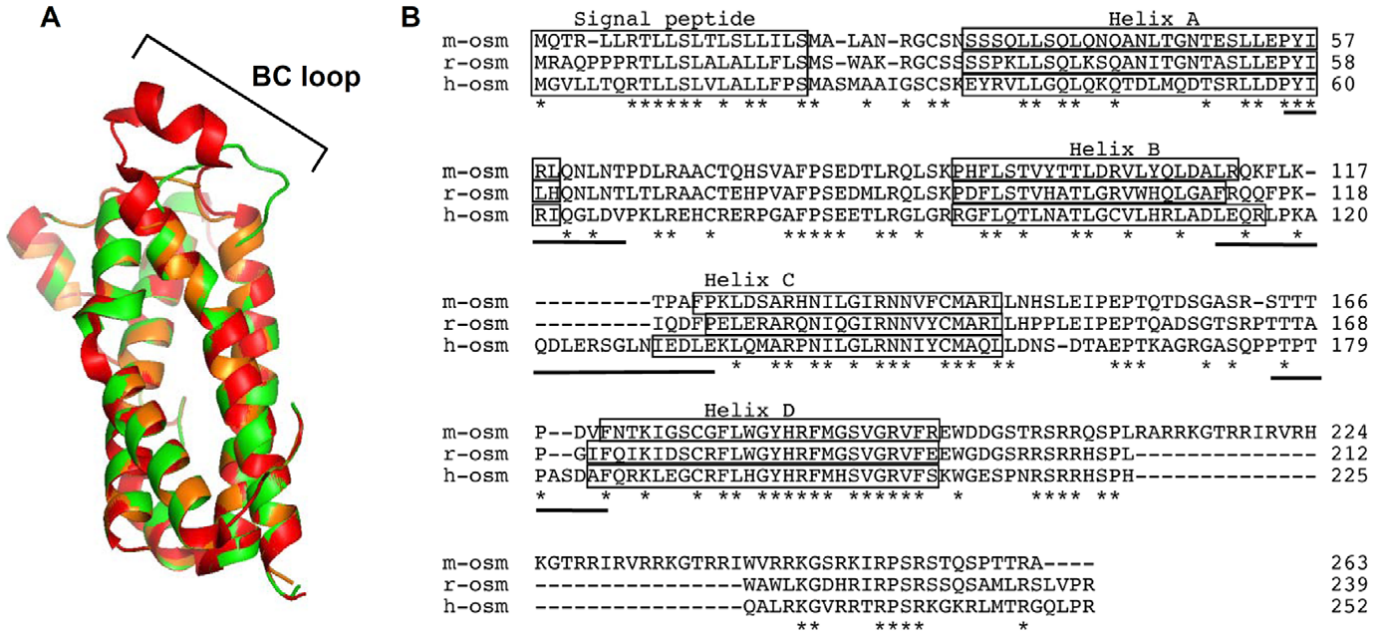
Differences in the BC loop of human, murine and rat OSM might be responsible for the divergent receptor usage. **A**, Model structure of mOSM (amino acids 25–205 of NP_001013383.1, orange) and rOSM (amino acids 26–207 of NP_001006962.1, green) using the solved crystal structure of human OSM (PDB entry code: 1EVS, red) as template. For molecular modeling and graphic representation the SWISS-MODEL-Server [49], [50], [51] and PyMOL (DeLano, W.L. (2002) The PyMOL Molecular Graphics System. DeLano Scientific, San Carlos, CA, USA) were used. **B**, Sequential alignment of murine, rat and human OSM. A fold recognition algorithm was used to generate the sequential alignment (ProHit package, ProCeryon Biosciences GmbH, Salzburg, Austria). Asterisks indicate identical amino acid residues in all three species. Boxes denote helical regions and bars indicate regions in the human OSM involved in the site 3 binding site.

In this study we characterized the rat OSM receptor system and to our surprise, rOSM is more homologous to hOSM than to mOSM in that it can signal also via both receptor complexes. Taken into consideration that rOSM and mOSM share 60.2% sequence identity and rOSM and hOSM only share 49% identity (EBLOSUM62 Matrix, Gap penalty: 10.0, Extend penalty: 0.5) this finding is rather surprising (Fig. 7B). However, analysis of the contact sites of LIFR utilizing cytokines like ciliary neurotrophic factor (CNTF) found surprisingly large interfaces between cytokine and receptor [43]. Regarding the cytokine, the so-called site 3 encompasses amino acid residues of the C-terminal A helix extending to the N-terminal AB loop, the BC loop and the C-terminal end of the CD loop reaching into the N-terminal D helix (Fig. 7B, underlined sequences). Sequence comparisons of the three OSM variants indicate that the BC loop appears to be most divergent between the species (Fig. 7B). Modeling the three OSM structures based on the solved crystal structure of hOSM reveals an extremely short BC loop in mOSM, while it is substantially longer in hOSM and rOSM (Fig. 7A). Whether this region is indeed important to allow high affinity binding to the LIFR has to be determined by future mutagenesis experiments. Upon successful identification of these amino acid residues the subsequent mutagenesis of mOSM might allow its conversion into a variant comparable to human OSM. Thereby the generation of a humanized mouse model might be possible in future to evaluate the physiological role of OSM.

## Materials and Methods

### Reagents, recombinant cytokines, cell lines and primary cells

Recombinant hOSM, rOSM and mIL-3 were purchased from Peprotech, mOSM from R&D Systems and hLIF from Sigma-Aldrich. Recombinant LIF-05 was prepared as described previously [35] and kindly provided by Prof. Dr. J. Heath (University of Birmingham, UK). JTC-27 rat and HepG2 human hepatoma cell lines were purchased from the DSMZ (Braunschweig, Germany), the Hepa 1c1c7 murine hepatoma cell line from Sigma-Aldrich. Primary rat dermal fibroblasts were obtained from PELOBiotech (Martinsried, Germany). All cell lines were cultured according to the suppliers' instructions at 5% CO_2_ and 37°C in water-saturated atmosphere. All media were obtained from Invitrogen and supplemented with 10% FCS (PAA). Ba/F3 cells stably expressing hgp130 and hOSMR [44] were kindly provided by Prof. Dr. J. Heath (University of Birmingham, UK) and primary human dermal fibroblasts [45] by Prof. Dr. J.M. Baron (Department of Dermatology and Allergology, RWTH Aachen University Hospital, Germany). Primary neonatal rat cardiac fibroblasts were prepared as described previously [46], but cultured in Medium 199 containing 10% FCS and kindly provided by Dr. K. Lorenz (Institute of Pharmacology and Toxicology, University of Würzburg, Germany).

### Cell lysis and Western blotting

Upon stimulation, cells were lysed in either ice-cold Triton X-100 lysis buffer containing 10 µl/ml Halt phosphatase inhibitor cocktail (Thermo Fisher Scientific Inc.) or 1 x Laemmli buffer (62.5 mM Tris-HCl, 10% glycerol, 2% sodium dodecyl sulfate (SDS), 0.0025% bromophenol blue and 5% β-mercaptoethanol, pH 6.8) as described previously [40]. Proteins were separated by 10% SDS-PAGE, followed by semi-dry Western blotting onto a PVDF-membrane (Whatman, GE Healthcare). Protein detection was conducted using the indicated antibodies and the enhanced chemiluminescence kit (Thermo Fisher Scientific Inc.) according to the manufacturers' instructions. Quantification of the chemiluminescence signal was carried out on the FluorChemQ using the AlphaView® software (ProteinSimple). Equal loading of the gel was verified by stripping the membrane in 62.5 mM Tris HCl (pH 6.7) containing 2% SDS and 100 mM β-mercaptoethanol at 70°C for 20 minutes and redetection with antibodies recognizing the protein irrespective of its phosphorylation status as well as by detection of tubulin.

### Antibodies

All antibodies were purchased from Cell Signaling Technology (New England Biolabs), with the exception of the antibodies detecting rat phospho-Tyr^694^-STAT5 (Signalway Antibody Co.), STAT5 (Santa Cruz Biotechnology), tubulin (Sigma-Aldrich), human and mouse OSMR (R&D Systems).

### Small interfering RNA (siRNA) transfection

For siRNA transfections, JTC-27 cells were seeded onto 6 cm dishes at a density of 3.0×10^5^ cells/dish and transfected using DharmaFECT 4 (Thermo Fisher Scientific Inc.) and 100 nM siRNA, while HepG2 and Hepa 1c1c7 were cultured on 6 wells at 2.0×10^5^ cells/well and transfected in Lipofectamine 2000 (Invitrogen) and 50 nM siRNA according to the manufacturers' instructions. Transfection was allowed to proceed for 5 hours at 37°C, before Opti-MEM containing FCS (f.c. 10%) was added. Cells were harvested after 28 hours (Hepa 1c1c7) or 48 hours (HepG2 and JTC-27). Rat and murine OSMR siRNAs (OnTargetPlus SMARTpools) were purchased from Dharmacon (Thermo Fisher Scientific Inc.), human OSMR siRNA from Ambion (Applied Biosystems) and nonsilencing control siRNA (AllStars Negative Control siRNA) from Qiagen.

### Semiquantitative and quantitative RT-PCR

After treatment of cells total RNA was isolated using the RNeasy kit (Qiagen) according to the manufacturer's instructions. 1 µg total RNA was used for cDNA synthesis using the OneStep RT-PCR kit (Qiagen) for semi-quantitative PCR or the Transcriptor First Strand cDNA Synthesis Kit from Roche Diagnostics for quantitative PCR. Real-time PCR was performed using the FastStart Universal SYBR Green Master (Rox) Kit (Roche Diagnostics) according to manufacturer's instructions. Specific primers were designed to be located across an exon/exon border. Primer sequences for semi-quantitative PCR are as follows: rat OSMR: forward 5′-ATATACCAGCGCTGGCCAGG-3′, reverse 5′-AATAGTCCGAGTTGGTGCGG-3′, rat GAPDH: forward 5′-TGATGACATCAAGAAGGTGG-3′, reverse 5′-TTACTCCTTGGAGGCCATGT-3′. The following primers were used for quantitative RT-PCR: rat OSMR: 5′-CCTTCATCAAGTGACCTTCCTT-3′, reverse 5′-GTAAAGGCTCCCCCAAGACT-3′ and rat GAPDH: forward 5′-TGGGAAGCTGGTCATCAAC-3′, reverse 5′-GCATCACCCCATTTGATGTT-3′. Quantification of -fold inductions over untreated samples was performed using the mathematical model described by Pfaffl [47].

### Construction of expression vectors

Standard cloning procedures were performed throughout. To generate tetracycline-inducible bidirectional promoter driven expression plasmids encoding the rgp130/rLIFR combination or the rgp130/rOSMR combination, we first cloned the cDNAs for each receptor using total RNA extractions from JTC-27 rat hepatoma cells. Upon reverse transcription, the cDNA was used to amplify the complete coding sequence of each receptor using specific primers containing restriction sites flanking the start or stop codon and the PCR Extender System (5 PRIME). The rgp130 amplicon was digested with *Age*I and *Not*I fast digest enzymes (Fermentas) for 30 minutes at 37°C. The rOSMR and rLIFR amplicons were digested with *Sbf*I and *Fse*I (New England Biolabs) for 4 hours at 37°C. After gel purification the fragments were ligated stepwise into the plasmid pBO (kindly provided by Dr. C. Haan, Luxembourg) which contains a tetracycline responsive bidirectional promoter to allow simultaneous transcription of two receptor cDNAs and a hygromycin B resistance cassette to allow selection of stable cell lines [48]. Thereby pBO-rgp130/rLIFR or pBO-rgp130/rOSMR was generated. The integrity of all constructs was verified by DNA sequence analyses (Eurofins MWG).

### Stable transfection of murine Ba/F3 cell line

The murine pre-B cell line Ba/F3 was first transfected with the 2.5 µg of the pTetON-neo plasmid (Clontech Laboratories Inc.) using the Nucleofector (Lonza) according to the manufacturer's instruction. A neomycin-resistant pool of cells was then transfected with 2.5 µg of the pBO-rgp130/rLIFR or the pBO-rgp130/rOSMR plasmid again using the Nucleofector. A hygromycin/neomycin-resistant pool of cells was selected and stimulated, upon overnight induction of the receptor expression with 0.5 µg/ml doxycycline, with 10 ng/ml hLIF, 20 ng/ml hOSM, mOSM or rOSM for 15 min.

### Proliferation assay

Proliferation of stably transfected Ba/F3-hgp130/hOSMR [44] in response to hOSM or rOSM was analyzed using the colorimetric proliferation assay reagent WST-1 from Clontech. Cells were seeded at a concentration of 1×10^4^ per 96-well and treated with indicated concentrations of hOSM or rOSM for 48 h. After the incubation period, 10 µl premixed WST-1 reagent were added to every well. After 4 h incubation at 5% CO_2_ and 37°C in water-saturated atmosphere, absorbance was measured at 450 nm and 660 nm using a Multiskan EX Microplate Photometer (Thermo Fisher Scientific Inc.). Proliferation assay results were calculated by subtracting the A_660_ value from the A_450_ value.

### Statistical analysis

All data are given as mean ± S.E.M. using a paired, two-tailed Student's t-test. A value of ***p***<0.05 was considered statistically significant. Densitometric units obtained for phosphorylated proteins were normalized to the loading control and either the rOSM- or the hOSM-stimulated sample was set to 100.

## Supporting Information

Figure S1 **Comparison of hLIF, hOSM, mOSM and rOSM activated signaling pathways in primary neonatal rat cardiac fibroblasts (NRCFB).** Cells were treated with 10 ng/ml hLIF, hOSM, mOSM or rOSM for 15 min. The phosphorylation levels of STAT1, STAT3 and ERK1/2 were detected via Western blot analysis. The blots were stripped and reprobed with antibodies recognizing the proteins irrespective of their phosphorylation status. Additionally, an α-tubulin loading control was included.
(TIF)
