# Supplementary figures and images for: Characterization of the Rat Oncostatin M Receptor Complex Which Resembles the Human, but Differs from the Murine Cytokine Receptor
Source: PLoS One. 2012 Aug 22;7(8):e43155. doi: 10.1371/journal.pone.0043155 (PMC3425591; doi:10.1371/journal.pone.0043155)

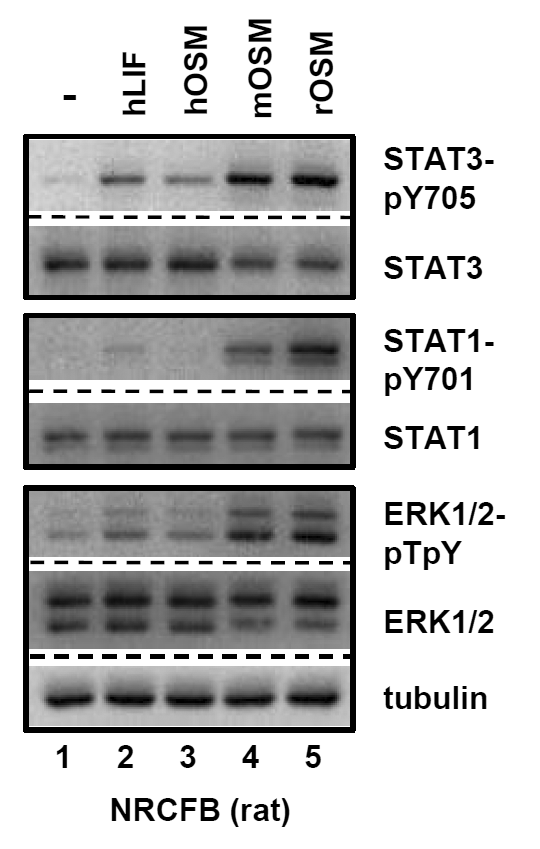

Supplement: Figure S1 — Comparison of hLIF, hOSM, mOSM and rOSM activated signaling pathways in primary neonatal rat cardiac fibroblasts (NRCFB). Cells were treated with 10 ng/ml hLIF, hOSM, mOSM or rOSM for 15 min. The phosphorylation levels of STAT1, STAT3 and ERK1/2 were detected via Western blot analysis. The blots were stripped and reprobed with antibodies recognizing the proteins irrespective of their phosphorylation status. Additionally, an α-tubulin loading control was included. (TIF) [file pone.0043155.s001.tif]
